# Retraction Note: Valproic acid attenuates intercellular adhesion molecule-1 and E-selectin through a chemokine ligand 5 dependent mechanism and subarachnoid hemorrhage induced vasospasm in a rat model

**DOI:** 10.1186/s12950-023-00370-x

**Published:** 2023-12-20

**Authors:** Chih-Zen Chang, Shu-Chuan Wu, Chih-Lung Lin, Aij-Lie Kwan

**Affiliations:** 1 Department of Surgery, Faculty of Medicine, School of Medicine, Kaohsiung Medical University, Kaohsiung, Taiwan; 2 Division of Neurosurgery, Department of Surgery, Kaohsiung Medical University Hospital, No.100, Tzyou 1st Road, Kaohsiung, Taiwan; 3 Department of Surgery, Kaohsiung Municipal Ta Tung Hospital, Kaohsiung, Taiwan

**Retraction Note: J Inflamm 12, 27 (2015)**

**https://doi.org/10.1186/s12950-015-0074-3**

The Editors-in-Chief have retracted this article. After publication, concerns were raised regarding the data presented in the figures, specifically:

- Fig. 1c appears highly similar to Fig. 1c of [1], Fig. 1b of [2] and Fig. 1c of [3].
- Fig. 1f appears highly similar to Fig. 1f of [3].
- Fig. 1c also appears highly similar to 1b in [4].
- The b-actin blots across all figures appear highly similar (rotated in Fig. 4), although they represent different groups.
- The b-actin blots also appear highly similar to b-actin in Fig. 3 and GAPDH in Fig. 4 of [5].
- Sham data are not presented in Figs. 2-4.
- SAH+V image is not included in Fig. 2.
- The error bars in the graphs appear unusually consistent.

The Editors-in-Chief therefore no longer have confidence in the presented data.

None of the authors have responded to any correspondence from the publisher about this retraction.
